# Extraocular Muscle Fixation to the Orbital Wall

**Published:** 2010-04

**Authors:** Zhale Rajavi

**Affiliations:** Ophthalmic Research Center, Imam Hossein Hospital, Shahid Beheshti University of Medical Sciences, Tehran, Iran

**Keywords:** Oculomotor Muscles, Orbit

## Abstract

The surgical results of severe or complex deviations such as those due to complete third nerve palsy, aberrant innervation of extraocular muscles (EOMs) and Duane syndrome are usually not completely successful. Herein, we describe the surgical technique of EOM fixation to the orbital wall. After a limbal or fornix based conjunctival incision, the related EOM is identified and dissected; the muscle insertion is sutured with non-absorbable sutures and detached from the sclera. The adjacent periosteum is exposed approximately 5 mm posterior to the orbital rim. The sutured muscle is then fixed to the orbital wall with two periosteal bites. The cut edges of the intermuscular membrane are closed over the sclera to avoid adherence of the muscle to the sclera. Finally the conjunctiva is reapproximated or recessed if necessary. This method of EOM inactivation completely eliminates all muscle forces from the globe and can provide better alignment in the above mentioned types of strabismus. The procedure is reversible and can be converted to other types of weakening operations if necessary.

## INTRODUCTION

Surgical alignment of complete oculomotor nerve palsy, large-angle sensory deprivation exotropia (XT), congenital or acquired aberrant innervation of extraocular muscles (EOMs), and specific types of Duane syndrome frequently yield unsatisfactory results. In many cases the patient requires multiple operations to maintain alignment in primary position.^1^

Common surgical methods for the treatment of complete third nerve palsy include: lateral rectus (LR) recession and medial rectus (MR) resection;^2^ free myectomy of the LR;^3^ superior oblique (SO) transposition to the superior rectus (SR) or MR^4^–^6^; globe fixation by an apically based periosteal flap^7^ or elastic silicone band^8^,^9^ or SO tendon^10^, with simultaneous LR recession; and medial transposition of the LR.^11^ These procedures usually result in under-correction or XT and may cause complications such as severe LR underaction, recurrence of XT due to secondary attachment of the LR to the globe, hypertropia, V-pattern, intraoperative hemorrhage, silicone band extrusion, globe perforation, postoperative lid edema and periocular inflammation.^1^

A new surgical technique of LR fixation to the adjacent orbital wall can completely eliminate LR muscle activity and help achieve better alignment.^12^,^13^ Advantages of this procedure include reduced risk of globe perforation, prevention of muscle attachment to the globle and recurrent XT, fewer number of operations, and reversibility of the procedure. Disadvantages of this new method include difficult technique, possible lacrimal gland damage and postoperative lid edema.^1^ Recently, satisfying results have been reported for this technique in congenital aberrant innervation of extraocular muscles and inferior oblique overaction as well.^14^,^15^ Herein, we describe the surgical technique of EOM fixation to the orbital wall.

## SURGICAL TECHNIQUE

**Figure 1:**
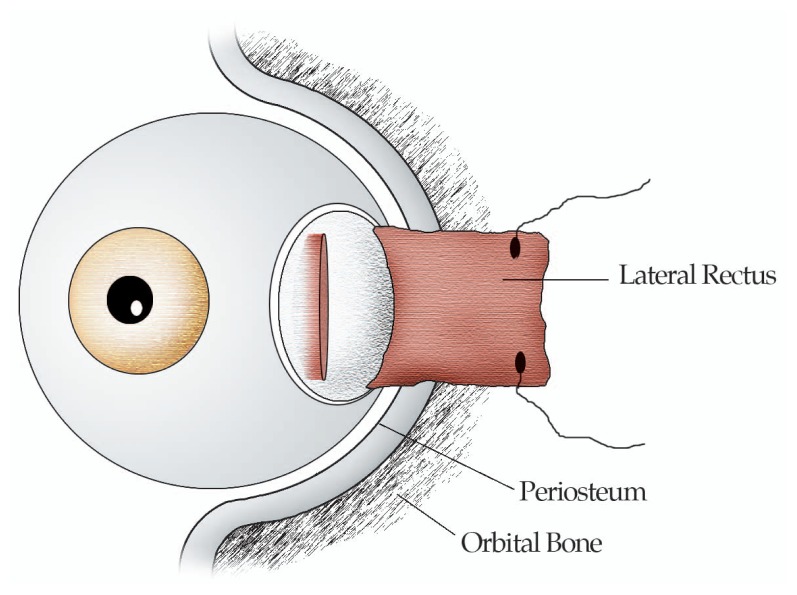
Detaching the lateral rectus from the sclera.

**Figure 2:**
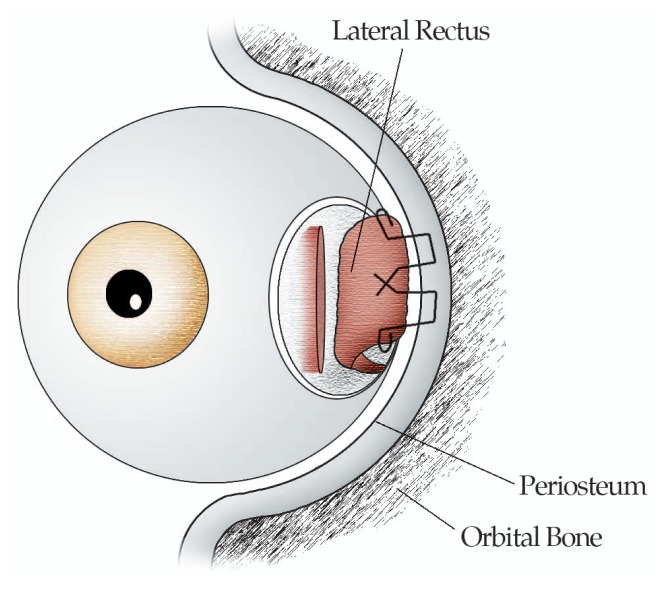
Attaching the lateral rectus to the periosteum.

Rectus muscle fixation to the orbital wall is performed in the following fashion. Under general anesthesia, a lid speculum is inserted and passive forced duction test (FDT) is performed. Then a 7-0 silk traction suture is placed at the limbal area adjacent to the planned rectus muscle and the globe is rotated in the opposite direction. A limbus or fornix based conjunctival incision is fashioned and the rectus muscle is dissected from the surrounding intermuscular membrane (IMM) and Tenon’s capsule, and isolated on a muscle hook. The insertion of the rectus muscle is sutured by non-absorbable 5-0 Ethicon sutures and detached from the globe using Westcott scissors. The adjacent periosteum, approximately 5 mm posterior to the orbital rim, is exposed outside the muscle cone and the sutured rectus muscle is fixed by 2 periosteal bites to the orbital wall. The cut edges of the IMM are sutured together over the sclera with non-absorbable sutures to avoid adherence of the muscle to the sclera. The conjunctival incision is closed by interrupted 6/0 polyglactin sutures and can be recessed if the conjunctiva is found to be short and tight.^1^,^12^ (Figures 1,2)

Because of the relationship between the SR and the levator muscle, such procedures entail the risk of post-operative superior lid position abnormalities; therefore the SR should be fixed to the medial rather than the superior orbital wall to avoid such complications.^1^ In case of esotropia following sixth nerve palsy, when the SR and inferior rectus (IR) are intact, transposition surgery is indicated instead of MR fixation to the medial orbital wall.^1^

Surgical stages for inferior oblique (IO) muscle fixation are as follows: after insertion of a lid speculum and evaluation of passive FDT, 7-0 silk traction sutures are placed at the temporal and inferior limbus, and the globe is rotated superonasally. An 8 mm long conjunctival incision is fashioned circumferentially at the inferior temporal fornix. The IO muscle is identified and isolated on a muscle hook and dissected from the surrounding connective tissue. The insertion of the IO muscle is clamped, detached and sutured by non-absorbable 5-0 Ethicon sutures. The periosteum just beneath the lateral orbital rim is exposed via blunt dissection using Westcott scissors; the sutured IO muscle is fixed to the lateral orbital wall using 2 periosteal bites. FDT is repeated at the end of the operation and the conjunctiva is closed with interrupted 6-0 polyglactin sutures.

The difference between pre- and postoperative deviation (with normal eye fixation), head posture at distance fixation, and ductions and versions (in scales ranging from −4 for lack of movement, to +4 for maximum motility) are the three most important criteria for evaluation of the results of such procedures.

## DISCUSSION

The extraocular muscles and their surrounding connective tissues regulate globe position in the orbital cavity. Contractions and relaxations of EOMs cause globe rotations. The surgical results of complete third nerve palsy, aberrant innervation of EOMs and Duane syndrome are usually unsatisfactory. Multiple operations, with a mean of 2.3 per patient, are required for achieving acceptable alignment in primary position.^1^

For treatment of complete third nerve palsy, Helveston suggested supramaximal recession and resection, in which the LR muscle was recessed 8–10 mm with double 80% marginal myotomy and the MR was resected (12–14 mm), however 50% of the patients required more than one procedure.^1^ MR resection, even in supramaximal amounts employed with this technique, is not very effective due to complete palsy and LR recession may be complicated by globe perforation and postoperative limitation in abduction. Supramaximal recession and resection can be more effective in severe sensory XT than in complete third nerve palsy, which is due to absence of MR palsy. Another procedure reported to be effective for complete third nerve palsy is transposition of the SO tendon to the SR combined with supramaximal LR recession, however this procedure may lead to hypertropia, V-pattern deviation, and limitations in infraduction.^1^,^4^–^6^ While the apically based periosteal flap provides a good tether for the globe, significant postoperative swelling and lacrimal sac damage have been reported as complications.^7^ Medial transposition of the LR, 4 mm superoposterior to the MR insertion is another new experimental method for treatment of complete third nerve palsy.^10^ Sato et al^3^ suggested free myectomy of the LR, although reattachment of the LR to the globe by fibrous tissue has been demonstrated by MRI. Transposition of the vertical recti is not indicated since they may also be paralyzed.

The most common reason for multiple surgeries in eyes with complete third nerve palsy is residual XT due to remaining LR force even after supramaximal recession. LR inactivation, by its fixation to the lateral orbital wall, is an effective method for eliminating all LR forces from the globe, therefore it can provide better alignment and prevent the complications mentioned above.^1^

Miswiring and co-contraction are common in cases with aberrant EOM innervation. Decision for surgery should be made individually. For example Velez,^1^ reported a patient with large angle hypotropia in adduction and large angle hypertropia in abduction; the SR and IR were fixed to the superior and inferior orbital walls eliminating vertical misalignments.^1^

In a specific kind of Duane Syndrome (type III) with severe limitation in adduction and abduction, co-contraction, retraction and paradoxical XT in attempted adduction; LR fixation to the lateral orbital wall improved all signs, and the severe limitation in abduction was corrected by augmented partial vertical transposition surgery.^1^,^13^

Recurrence of IO overaction after weakening procedures for this muscle has been reported in 15 to 100% of cases. Reattachment of the IO to the globe is the main reason for this phenomenon. Total extirpation and nasal myectomy have been recommended for re-operation in these cases, but this may result in mydriasis and IO underaction. The technique of IO fixation to the lateral orbital wall has a profound weakening effect on the IO muscle with no complication of mydriasis. This procedure is reversible and can be converted to other types of weakening procedures if needed.^15^
